# A Potential Daidzein Derivative Enhances Cytotoxicity of Epirubicin on Human Colon Adenocarcinoma Caco-2 Cells

**DOI:** 10.3390/ijms14010158

**Published:** 2012-12-21

**Authors:** Yu-Li Lo

**Affiliations:** Department of Biological Sciences and Technology, National University of Tainan, Tainan 700, Taiwan; E-Mail: yulilo@mail.nutn.edu.tw; Tel.: +886-6-260-6123; Fax: +886-6-260-6153

**Keywords:** multidrug resistance, reactive oxygen species, apoptosis, epirubicin, 8-hydroxydaidzein, isoflavones

## Abstract

In this study, we evaluated the effects of 8-hydroxydaidzein (8HD), an isoflavone isolated from fermented soy germ koji, and epirubicin (Epi), an antineoplastic agent, on the production of reactive oxygen species (ROS). We subsequently correlated the ROS levels to the anticancer mechanisms of Epi and 8HD in human colon adenocarcinoma Caco-2 cells. 8HD enhanced cytotoxicity of Epi and generated a synergistic effect. Epi and/or 8HD treatments increased the hydrogen peroxide and superoxide levels. Combined treatment markedly decreased mRNA expression levels of multidrug resistance protein 1 (MDR1), MDR-associated protein (MRP) 1, and MRP2. 8HD significantly intensified Epi intracellular accumulation in Caco-2 cells. 8HD and/or Epi-induced apoptosis, as indicated by the reduced mitochondrial membrane potential and increased sub-G1 phase in cell cycle. Moreover, 8HD and Epi significantly enhanced the mRNA expressions of Bax, p53, caspases-3, -8, and -9. To our best knowledge, this study verifies for the first time that 8HD effectively circumvents MDR in Caco-2 cells through the ROS-dependent inhibition of efflux transporters and p53-mediated activation of both death receptor and mitochondrial pathways of apoptosis. Our findings of 8HD shed light on the future search for potential biotransformed isoflavones to intensify the cytotoxicity of anticancer drugs through simultaneous reversal of pump and nonpump resistance.

## 1. Introduction

Combining different chemotherapeutics offer the benefit of having a second compound that enhances the anticancer efficacy of the primary agent, but decreases overall toxicity. Therefore, validation of natural compounds, such as isoflavones, as adjuvant agents to the current antineoplastic agents has become a potent chemotherapy strategy [1].

Colon cancer is one of the most common cancer-related deaths throughout the world [2]. Frequent development of multidrug resistance (MDR) hampers the efficacy of available anticancer drugs for colorectal cancer treatment [3]. MDR is the intrinsic and acquired resistance of cancer cells to chemotherapy. Multiple mechanisms contribute to chemoresistance, eventually leading to failure of cancer chemotherapy. The overexpression of P-glycoprotein (P-gp, product of *MDR1*) and MDR-associated proteins (MRPs), such as MRP1 and MRP2, which actively efflux anticancer drugs is one of the primary mechanisms of MDR [4]. However, inhibitors of drug efflux pumps in conventional MDR reversal strategy usually do not efficiently increase the sensitivity of chemotherapeutic drugs because of the complex mechanisms leading to MDR [4,5]. This failure is mainly caused by the activation of an antiapoptotic survival defense system, such as Bcl-2 protein [6]. Therefore, inhibiting Bcl-2 and other antiapoptosis-related proteins, as well as inducing apoptosis-related proteins such as Bax, may antagonize MDR and enhance the therapeutic efficacy of antineoplastic agents [5,6].

ATPase of P-gp is primarily dependent on ATP produced in mitochondrial oxidative phosphorylation, thus accelerating formation of toxic reactive oxygen species (ROS) [7]. In addition, ROS generation is closely related to P-gp and/or MRP regulation [8,9]. Furthermore, ROS may initiate apoptosis in various ways, such as by leading to mitochondrial permeability transition and the release of cytochrome c, a process inhibited by Bcl-2 [10]. Additionally, anticancer drugs increase oxidant stress leading to cytotoxicity enhancement in tumor cells [11].

**Figure 1:**
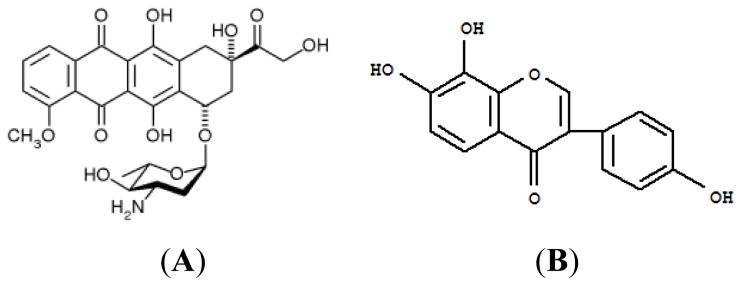
Chemical structure of (**A**) epirubicin (Epi) and (**B**) 8-hydroxydaidzein (8HD).

Epirubicin (Epi) is an anthracycline antineoplastic agent and a substrate of P-gp, MRP1, and MRP2 [5,12]. This study used Epi as a model anticancer drug, and its chemical structure is shown in Figure 1A. This compound induces apoptosis through the intrinsic mitochondrial signaling pathway [5,13].

7,8,4′-Trihydroxyisoflavone (8-hydroxydaidzein; 8HD) can be converted from daidzein in human and rat liver [14]. This compound has also been successfully prepared through biotransformation of daidzein in the soy germ fermented by Aspergillus oryzae (*A. oryzae*) [15–17]. The chemical structure of 8HD is shown in Figure 1B. A number of reports have revealed that 8HD exhibits various biological properties including radical-scavenging, cardioprotective, antioxidative, antiproliferative, and tyrosine-inhibitory activities [15,18–23]. This compound showed antimutagenic and melanogenesis-inhibitory activities *in vitro*, and skin-whitening activity *in vivo* [17,24]. 8-HD was easily absorbed into rats after oral administration [25]. Free 8-HD is changed into its glucuronide and/or sulfate in liver, whereas the conjugated 8-HD is released into systemic circulation [25]. This finding suggests that 8-HD exhibits its antioxidant activity both *in vitro* and *in vivo* [25]. In addition, its precursor daidzein increased intracellular drug levels by modulating P-gp and MRP function in various cell lines and mice [26–28]. However, there is no report regarding the effect of 8HD on MDR modulation. Therefore, this is the first study to show that 8HD reverses MDR transporters through production of ROS.

Furthermore, 8HD exhibited a remarkable antiproliferative activity toward human promyelocytic leukemia cells [23]. However, no paper to date has reported a significant apoptosis-inducing effect of 8HD. This compound marginally activated cancer cell death in the prostate cancer cells [20]. On the contrary, daidzein triggers apoptosis in different cancer types [28,29] and inhibits tumor growth [28]. Moreover, daidzein inhibits the proliferation and induces cell cycle arrest at G0/G1 and G2/M phases through a caspase-3-mediated apoptosis pathway in various cancer cells [1,30–32]. It is thus a pioneer study to correlate the relationship between the ROS levels and apoptosis-inducing mechanisms provoked by 8HD and/or Epi.

In this study, we suggest for the first time that 8HD enhances Epi’s cytotoxicity through the ROS-dependent inhibition of MDR transporters (e.g., P-gp, MRP1, and MRP2) and p53-mediated activation of both death receptor and mitochondrial pathways of apoptosis (e.g., Bax and caspases) in human colon adenocarcinoma Caco-2 cells. P-gp and MRPs are present in Caco-2 cells [33].

## 2. Results and Discussion

### 2.1. Results

#### 2.1.1. Combined Epi and 8HD Treatment Significantly Increased Epi Cytotoxicity and Caused Synergy

**Figure 2:**
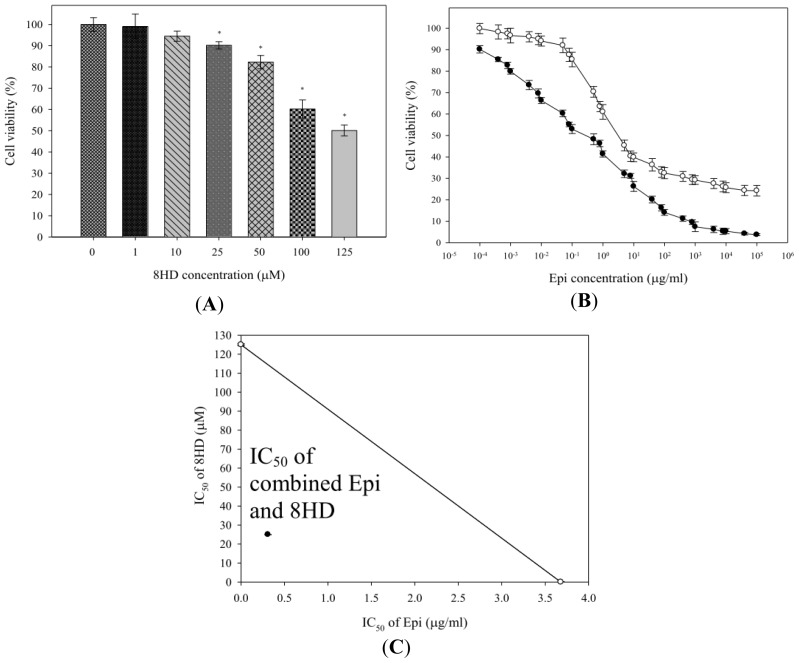
(**A**) The effect of 8HD on the growth of human colon adenocarcinoma Caco-2 cells. Cells were treated with 8HD at the concentrations of 0, 10, 25, 50, and 100 μM for 48 h. * *p* < 0.05 compared to the control. (**B**) The effect of 8HD on the cytotoxicity of Epi in Caco-2 cells; ○: Epi alone; ●: Epi plus 8HD (25 μM). (**C**) Isobologram analysis for the combination of Epi and 8HD; ○: Epi or 8HD alone; ●: Epi plus 8HD. Data are presented as means ± standard deviation (SD) from three independent experiments.

The relative cell viability (%) of Caco-2 cells treated with various 8HD concentrations (0, 10, 25, 50, and 100 μM) for 48 h was evaluated using MTT (3-(4,5-dimethylthiazol-2-yl)-2,5-diphenyl tetrazolium bromide) assay and is shown in Figure 2A. The viability of cells was 90.19% ± 1.70% 48 h post-incubation with 25 μM of 8HD, in contrast to the viability of 94.48% ± 2.41%, 82.31% ± 3.10%, and 60.23% ± 4.28% post-treatment with 10, 50, and 100 μM of 8HD, respectively. Hence, we chose 25 μM of 8HD for the combination study with Epi (10^−4^ μg/mL to 10^5^ μg/mL), as shown in Figure 2B, given that our aim was to develop 8HD as an adjuvant to intensify Epi potency. Epi potency in cell-growth inhibition was expressed as IC_50_ value, defined as the drug concentration necessary to inhibit cell growth by 50%. The mean IC_50_ value for combined Epi and 8HD treatment was 0.31 ± 0.05 μg/mL, significantly lower than that of Epi alone (3.68 ± 0.23 μg/mL; *p* < 0.05). This combination intensified Epi cytotoxicity and allowed for reduced Epi dosage.

Synergistic effect of combined 8HD and Epi was studied by isobologram analysis. IC_50_ of Epi was demonstrated in the *X*-axis of Figure 2C, whereas IC_50_ of 8HD was shown in the *Y*-axis. The diagonal line connecting the IC_50_ values of 8HD and Epi represents an additive isobole. However, the IC_50_ values of combined 8HD and Epi were far below the linear line, suggesting that combined 8HD and Epi treatment may produce a synergistic cytotoxicity in reduction of cell viability (Figure 2C). The CI was used to evaluate the synergistic effect. The CI was calculated from the IC_50_ values of 8HD and Epi as: (0.31/3.68) + (25/125) = 0.28, indicating a synergistic action of a combination of 8HD and Epi in Caco-2 cells.

#### 2.1.2. Combined 8HD and Epi Treatment Induced ROS Production

**Figure 3:**
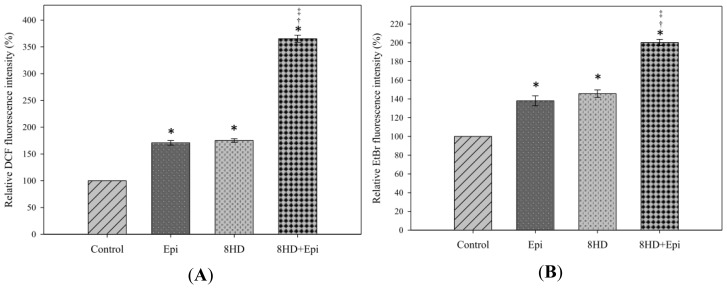
The effect of 8HD (25 μM) and/or Epi (0.3 μg/mL) on hydrogen peroxide and superoxide production. Cells were incubated with 8HD and/or Epi for 48 h. (**A**) Mean dichlorofluorescein (DCF) fluorescence intensity of cell control was normalized as 100%. (**B**) Mean ethidium bromide (EtBr) fluorescence intensity of cell control was normalized as 100%. Mean fluorescence intensity of treatment with Epi, 8HD, and Epi plus 8HD was normalized relative to the cell control. Data are presented as means ± SD from three independent experiments. * *p* < 0.05 compared to the control; † *p* < 0.05 compared to 8HD; ‡ *p* < 0.05 compared to Epi.

Intracellular ROS generation was determined using two probes, 2′,7′-dichlorofluorescein diacetate (DCFH-DA) and dihydroethidium (DHE). DCFH-DA is changed into fluorescent dichlorofluorescein (DCF) by H_2_O_2_. The relative DCF fluorescence intensity measurement shows that Epi exhibited similar H_2_O_2_ production to that of 8HD (Figure 3A). The intracellular H_2_O_2_ level was significantly higher after combined treatment compared with the levels of single administration of Epi or 8HD (*p* < 0.05).

DHE dehydrogenates to form the fluorescent ethidium bromide (EtBr) when reacting with O_2_^−^. Caco-2 cells treated with independent 8HD or Epi showed significant changes in O_2_^−^ generation compared with control (Figure 3B). Furthermore, the relative EtBr fluorescence intensity showed that combining 8HD with Epi increased O_2_^−^ production to 200.26% ± 3.37%.

#### 2.1.3. Combined 8HD and Epi Treatment Significantly Inhibited mRNA Expression Levels of P-gp, MRP1, and MRP2

**Figure 4:**
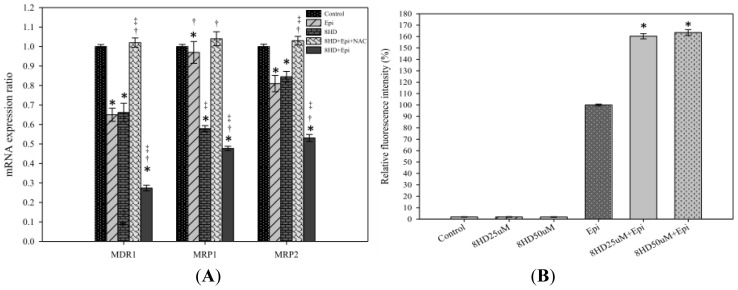
(**A**) The effect of treatment with medium (control), Epi (0.3 μg/mL), 8HD (25 μM), 8HD plus Epi plus N-acetyl cysteine (NAC; 5 mM), and Epi plus 8HD for 48 h on the expression ratio of the MDR pump-related genes encoding MDR1, MRP1, and MRP2. * *p* < 0.05 compared to the control; † *p* < 0.05 compared to 8HD; ‡ *p* < 0.05 compared to Epi. (**B**) The effect of 8HD (25 and 50 μM) on the intracellular accumulation of Epi (0.3 μg/mL). Mean fluorescence intensity of Epi was normalized as 100%. Mean fluorescence intensity of Epi plus 8HD (25 or 50 μM) was normalized relative to the Epi. * *p* < 0.05 compared to Epi; † *p* < 0.05 compared to 8HD (25 μM) plus Epi. The mean fluorescence intensity of 25 and 50 μM of 8HD are also shown here to demonstrate that the auto-fluorescence of 8HD was negligible. Data are presented as means ± SD from three independent experiments.

Real-time PCR primer sequences are listed in Table 1. Corresponding mRNA expression levels of MDR1 and MRP2 were significantly decreased by Epi (*p* < 0.05), whereas those of MDR1, MRP1, and MRP2 (*p* < 0.05) were remarkably reduced by 8HD (Figure 4A). mRNA expression ratio of MDR1, MRP1, and MRP2 after combined treatment was markedly lower than that of control (*p* < 0.05) and significantly lower than those of Epi or 8HD alone (both *p* < 0.05), implying that MDR1-, MRP1-, or MRP2-mediated pump resistance was efficiently reversed by the combined treatment (Figure 4A). In addition, we found that the ROS scavenger *N*-acetyl cysteine (NAC; 5 mM) significantly abrogated the reduction effect of 8HD and Epi on the mRNA expression of MDR1, MRP1, and MRP2 (Figure 4A). This result supports the involvement of ROS in 8HD- and Epi-mediated modulation of the MDR transporter expression.

#### 2.1.4. 8HD Significantly Increased the Intracellular Epi Accumulation in Caco-2 Cells

Both concentrations of 8HD significantly increased the intracellular Epi accumulation in Caco-2 cells (*p* < 0.05; Figure 4B). We normalized the fluorescence intensity of Epi to 100%. The relative fluorescence intensity (%) of Epi combined with 25 μM of 8HD increased to 160.30% ± 2.26%. Increasing 8HD concentration from 25 to 50 μM showed no further enhancement in Epi retention (*p* > 0.05; Figure 4B).

#### 2.1.5. Combined 8HD with Epi Decreased Mitochondrial Membrane Potential

**Figure 5:**
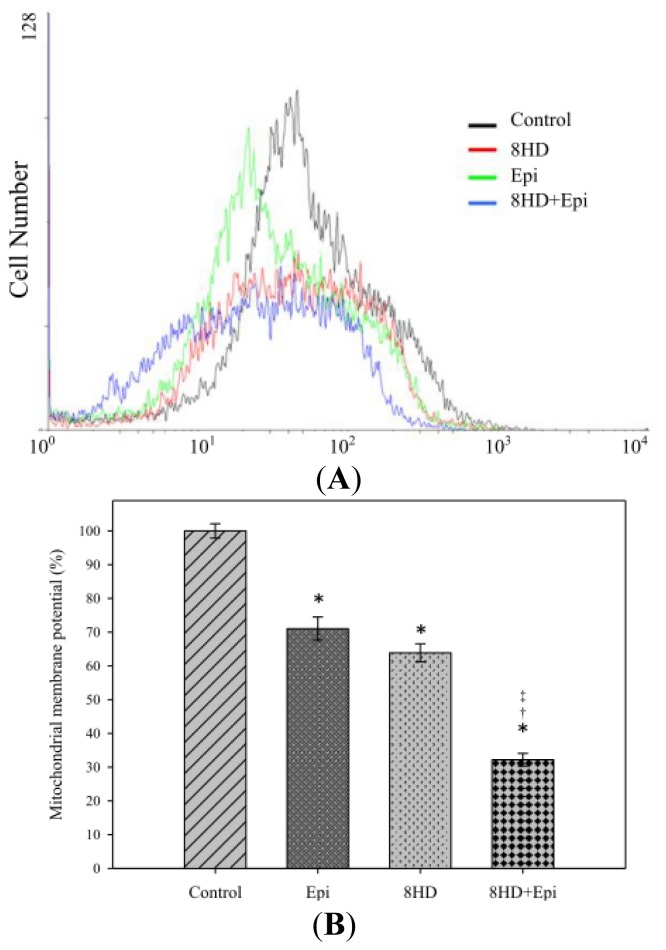

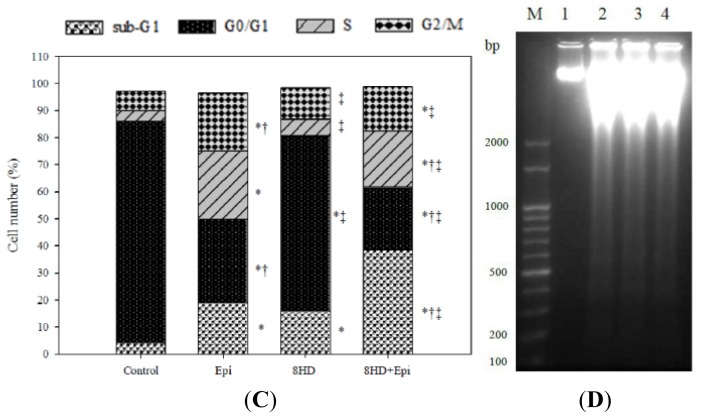
The effect of 8HD and/or Epi on mitochondrial membrane potential in Caco-2 cells. Cells were treated with 8HD (25 μM) and/or Epi (0.3 μg/mL) for 48 h and then stained with 3,3′-dihexyloxacarbocyanine iodide (DiOC_6_) and incubated at 37 °C for 60 min. (**A**) Three-dimensional view of cell number *versus* fluorescence intensity of DiOC_6_ in Caco-2 cells. (**B**) Mean DiOC_6_ fluorescence intensity of cell control was normalized as 100%. Mean fluorescence intensity of treatment with Epi, 8HD, and Epi plus 8HD was normalized relative to the cell control. (**C**) Analysis of cell number percent of each cell cycle phase relative to total phases using a flow cytometer. Data are presented as means ± SD from three independent experiments. * *p* < 0.05 compared to the control; † *p* < 0.05 compared to 8HD; ‡ *p* < 0.05 compared to Epi. (**D**) DNA fragmentation effect of 8HD and/or Epi on Caco-2 cells. DNA was resolved by electrophoresis on 1.2% agarose gel and then visualized by SYBR safe staining. Line M: Marker, Line 1: ontrol, Line 2: Epi (0.3 μg/mL), Line 3: 8HD (25 μM), Line 4: 8HD + Epi.

The mitochondrial membrane potential (ΔΨm) can be measured by a cationic lipophilic fluorochrome 3,3′-dihexyloxacarbocyanine iodide (DiOC_6_). Cells treated with 25 μM of 8HD showed a decrease in ΔΨm (control: 100%; 8HD: 63.89% ± 2.68%; Figure 5A,B). We also found that Epi caused a reduction in ΔΨm (Epi: 71.03% ± 3.46%). When we combined 8HD with Epi, Ψm is further decreased to 32.18% ± 1.88%, which was significantly lower than the treatment of either 8HD or Epi alone (Figure 5A,B).

#### 2.1.6. Combined 8HD and Epi Treatment Significantly Increased Sub-G1 Phase of Caco-2 Cell Cycle

Percentages of the sub-G1 phase of Caco-2 cells, corresponding to the proportions of apoptotic cells, significantly increased after 8HD and/or Epi treatments (Figure 5C). The percentage of the sub-G1 phase of cells treated with 8HD and Epi (*p* < 0.05) was significantly higher than that of cells treated with 8HD or Epi. This result suggests that the combined treatment induced more cells to undergo apoptosis, and that 8HD might increase the ability of Epi to induce apoptosis in Caco-2 cells. We also found a mild increase in the G2/M phase for all treatments compared with the control (Figure 5C).

#### 2.1.7. 8HD and/or Epi-Induced DNA Fragmentation

DNA fragmentation is a molecular biological characteristic of apoptosis, during which chromatin is digested by endonuclease. Thus, the apoptotic effect of 8HD and/or Epi on Caco-2 cells was further examined using agarose gel electrophoresis. The results revealed that 8HD, Epi alone, and 8HD combined with Epi all formed a ladder pattern with multiples of approximately 100 bp to 200 bp DNA fragments (Figure 5D). Caco-2 cells exposed to 8HD (25 μM) and/or Epi (0.3 μg/mL) for 48 h also exhibited cell shrinkage and bright green areas of condensed or fragmented chromatin in the nucleus (microscopic data not shown), as examined using acridine orange staining and the inverted fluorescence microscopy. Thus, we further confirmed that the cytotoxic effect of 8HD and/or Epi on Caco-2 cells was mediated through apoptosis induction.

#### 2.1.8. Combined 8HD with Epi-Modulated mRNA Expression Levels of Bax, Bcl-2, Caspases, and p53

**Figure 6:**
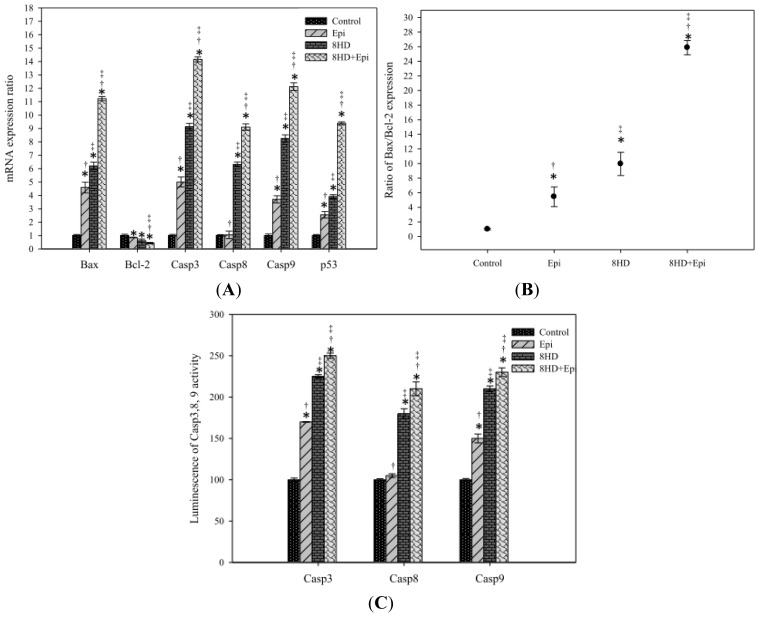
(**A**) The effect of 8HD and/or Epi on the expression ratio of the apoptosis-related genes encoding Bax, Bcl-2, caspase 3 (Casp3), caspase 8 (Casp8), caspase 9 (Casp9), and p53 in Caco-2 cells as determined by real-time PCR. (**B**) The effect of 8HD and/or Epi on the Bax:Bcl-2 ratios of mRNA expression. (**C**) The effect of 8HD and/or Epi on Casp3, 8, and 9 activities as recorded the luminescence using a luminometer. Cells were treated with the 8HD and/or Epi for 48 h. Data are presented as means ± SD from three independent experiments. * *p* < 0.05 compared to the control; † *p* < 0.05 compared to 8HD; ‡ *p* < 0.05 compared to Epi.

Treatments of 8HD or Epi alone significantly increased the corresponding mRNA levels of caspases-3, -8, and -9, as well as p53 and Bax (Figure 6A; *p* < 0.05). Combined Epi and 8HD resulted in significantly (*p* < 0.05) higher levels of caspases-3, -8, and -9, as well as p53 and Bax expression than those of the treatments of 8HD or Epi alone (all with *p* < 0.05). Moreover, all these treatments remarkably increased the Bax-to-Bcl-2 ratio and showed significant reduction on Bcl-2 expression (*p* < 0.05). Combining 8HD and Epi resulted in further enhancement of the Bax-to-Bcl-2 ratio (Figure 6B; *p* < 0.05). This finding suggests that 8HD might increase the sensitivity of apoptosis induced by Epi and make Caco-2 cells more susceptible to undergo apoptosis.

#### 2.1.9. Combined 8HD and Epi Treatment Increased Caspases-3, -8, and -9 Activities

We examined the activities of caspases-3, -8, and -9 induced by 8HD with or without Epi to determine the apoptotic pathway involved. The activities of these caspases were increased by 8HD or Epi alone (*p* < 0.05) and further enhanced by the combined treatment (Figure 6C; *p* < 0.05).

### 2.2. Discussion

**Figure 7:**
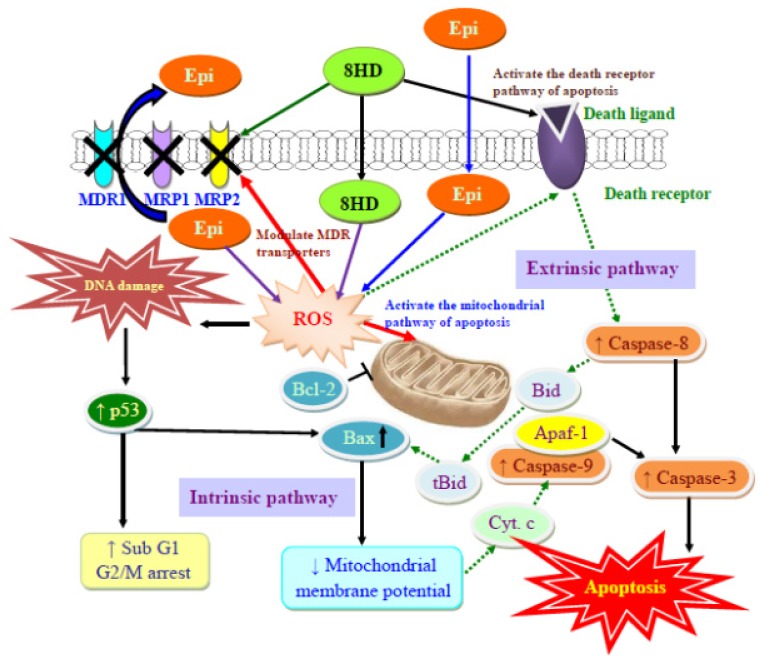
The proposed pathways for reversing MDR transporters and inducing apoptosis in Caco-2 cells by 8HD and/or Epi. Apaf-1 is apoptotic protease activating factor 1. Cyto. c is cytochrome c.

Epi, a substrate of P-gp, MRP1, and MRP2 [5,12], was selected as a model anticancer drug in this study. The fermented product 8HD exhibits cytotoxic effect on Caco-2 cells (Figure 2A). Thus, this compound is used as an adjuvant with Epi in Caco-2 cells to intensify potency of chemotherapy. An increase in ROS levels, including H_2_O_2_ and O_2_^−^ through co-incubation with 8HD (Figure 3) enhanced Epi cytotoxicity (Figure 2B) and generated a synergistic effect (Figure 2C). Previous investigations have suggested that daidzein, the precursor of 8HD, may exhibit multiple anticancer effects, including proliferation inhibition, P-gp and MRP suppression, and apoptosis induction [1,26–28,30,34]. It is worth stressing that the goal of the present work was to evaluate the effect of ROS production after 8HD and/or Epi treatments on triggering the cellular mechanisms of inhibiting efflux transporter-related MDR and inducing apoptosis. The proposed pathways for reversing pump and non-pump MDR in Caco-2 cells are shown in Figure 7. The detailed mechanisms are discussed as follows.

Intracellular ROS (iROS) have been implicated in P-gp and MRP modulation. ROS might act as negative regulators of P-gp expression [8,9]. An increase in iROS levels decreases P-gp expression, as well as reduces efflux of the P-gp substrate doxorubicin in prostate adenocarcinoma cells [9,35]. However, contradictory studies have demonstrated that high concentrations of ROS confer oxidative stress and enhance P-gp and MRP expressions [11,36]. These conflicting results on ROS levels and expression of MDR transporters inspired our current investigation.

The relationship between the effect of 8HD on ROS production and regulation of P-gp and MRPs has not been reported. In the present study, Epi-related pump resistance was reversed in various degrees by the addition of 8HD through the suppression of P-gp and MRPs. Accordingly, daidzein, the precursor of 8HD, increased intracellular drug levels by modulating P-gp and MRP functions in human cervical carcinoma cells and mice [26,34]. Daidzein was determined as a P-gp/MRP1 inhibitor, significantly increasing the uptake of [(3)H]-atazanavir (ATV) (a P-gp, MRP, and hOATP substrate) in human leukemia cells [27]. In addition, transport of conjugated daidzein is inhibited by the MRP inhibitor leukotriene C4 [24]. Daidzein also inhibits MRP1-, MRP4-, and MRP5-mediated transport and modulates MDR [37]. With these precedents in mind, we suggested that 8HD decreased mRNA expressions of functional MDR transporters (Figure 4A), which might reduce epirubicin efflux and enhance epirubicin accumulation (Figure 4B). The functional involvement of 8HD in reversing MDR transporter mediated Epi resistance was thus verified. We suggest that 8HD might function as a transporter substrate and thus compete with anticancer drug extrusion.

Our study demonstrates that 8HD and/or Epi enhanced ROS production, but reduced the expression of MDR1, MRP1, and/or MRP2, in agreement with other studies regarding the negative correlation between ROS production and MDR regulation [8,9,35]. In addition, we found that the ROS scavenger, NAC, one precursor of intracellular glutathione, abrogated the modulation effect of 8HD and Epi on the MDR transporter expression (Figure 4A). NAC might abolish the negative regulation of P-gp through the inhibition of c-Jun-*N*-terminal kinase (JNK) signaling pathway [38]. We thus verify that the decrease of MDR1 and MRP1 by 8HD and/or Epi through ROS production circumvented the pump resistance. However, the complicated regulation requires further clarification in the signaling pathway.

Intracellular ROS is likewise involved in apoptosis regulation. Potentiation of oxidative stress is closely linked to apoptotic response induced by a number of antineoplastic agents [11]. Interestingly, doxorubicin, an anthracycline anticancer drug and Epi anomer, induced tumor necrosis factor-alpha (TNF-alpha) that caused ROS generation, leading to mitochondrial dysfunction and apoptosis [11]. Furthermore, ROS played a key role in the cytotoxic effect of genistein on diverse cell lines [39,40]. Oxidative stress is thus confirmed as critical in apoptosis induction, acting as an upstream signaling initiator to trigger programmed cell death in different cell lines.

However, the relationship between the effect of 8HD on ROS production and apoptosis regulation has not been discussed in the literature. 8HD (7,8,4′-trihydroxyisoflavone) can be biotransformed from daidzein (4′,7-dihydroxyisoflavone), and thus 8HD may induce cancer cell death in mechanisms similar to those of daidzein. Daidzein triggered ROS generation, inhibited cell proliferation and caused significant cell cycle arrest in G1 and G2/M, and induced apoptosis through the mitochondrial caspase-dependent pathway in human cervical cancer, breast cancer, and murine neuroblastoma cells [1,30–32,41]. 8HD showed antioxidative and antiproliferative activities in human promyelocytic leukemia cells [18]. This compound also demonstrated antimutagenic activity and inhibitory activity against tyrosinase [24]. 8HD was verified as a potent suicide substrate of mushroom tyrosinase and showed *in vitro* cellular tyrosinase and melanogenesis inhibitory activities in mouse melanoma cells and *in vivo* skin-whitening activity in human volunteers [17]. In addition, this compound possessed DPPH-radical scavenging activity [19]. However, 8HD did not significantly induce apoptosis associated with changes in cell cycle distribution and caspase 3 activation in prostatic cancer cells [20].

With these precedents in mind, our current research clearly shows that 8HD and/or Epi initiated the central cell death signal by overexpressing p53, subsequently triggering the activated proapoptotic proteins, such as Bax. p53 played a key role in promoting cell apoptosis through a positive regulation of Bax and a negative regulation of Bcl-2 [42]. In addition, p53 caused arrest of cell cycle in G_1_ and G_2_/M by regulating genes such as *p21_WAF1_* [43]. An increase in the percentage of sub-G_1_ (apoptosis) and G_2_/M (completed DNA replication) fractions of the Caco-2 cell cycle (Figure 5C) also implies that checkpoints are initiated by oxidative stress so that cell cycles are paused for DNA repair and thus prevented cell progression from G_2_ into mitosis. Furthermore, it has been reported that the G_1_/S checkpoint suppresses the replication of the damaged DNA and that the G_2_/M checkpoint stops a cell from entering the M phase for a broken genome [40,44]. If the DNA damage cannot be repaired, the ROS produced by 8HD and/or Epi then increased the Bax/Bcl-2 ratio of the proapoptotic and antiapoptotic Bcl-2 family proteins. These changes were essential in opening the mitochondrial permeability transition pore, disruption of mitochondrial membrane potential, and activation of caspase-9 (Figures 5A and 6).

Two major apoptotic signaling pathways exist, namely, intrinsic or mitochondrial pathway and extrinsic or death receptor pathway. Caspase-8 is one of the initiator caspases for the death receptor pathway, whereas caspase-9 belongs to the initiator caspases for the mitochondrial pathway. Caspase-3 is a downstream effector caspase shared by both pathways [5,42]. In this study, independent Epi treatment significantly affected the caspase-9 and -3 expressions, but had only a marginal effect on caspase-8. This finding was in agreement with previous studies [5,13], confirming that Epi induces the mitochondrial apoptotic pathway. However, incubating Caco-2 cells with 8HD alone or combined with Epi significantly increased the expression levels of caspases-3, -8, and -9, as well as the activities of corresponding caspases in Caco-2 cells. This result suggested that both mitochondrial and death receptor pathways are involved in 8HD-mediated apoptosis. The caspase family of cystein proteases subsequently mediated a series of morphological changes, including chromatin condensation (data not shown) and DNA fragmentation (Figure 5D). A recent study has revealed that gomisin N, a lignan isolated from *Schisandra chinensis*, enhanced apoptosis induced by TNF-related apoptosis-inducing ligand (TRAIL) through ROS-mediated upregulation of death receptor 4 (DR4) and DR5, causing subsequent activation of caspases-3 and -8 [45]. We speculate that independent 8HD or its combination with Epi may also promote apoptosis mediated by TNF family proteins, such as Fas and TRAIL, through the caspase cascade, including caspases-3 and -8. Whether such a similar signaling pathway is involved needs further examination. To the best of our knowledge, we demonstrate for the first time that 8HD and Epi changed ROS levels; modulated the expression and function of efflux transporters; and induced apoptosis through both the intrinsic and extrinsic pathways in Caco-2 cells. We verified, at least in part, that 8HD and/or Epi activated the crosstalk among ROS levels, MDR transporter modulation, and apoptosis regulation in Caco-2 cells. The proposed schematic diagram for the possible underlying molecular mechanisms is shown in Figure 7.

## 3. Experimental Section

### 3.1. Materials

Epi (Pharmorubicin) was purchased from Pfizer Inc. (New York, NY, USA). All cell culture media and reagents were purchased from Gibco BRL (Grand Island, NY, USA) or Hyclone (Logan, UT, USA). Most of the other chemical reagents were purchased from either Merck (Darmstadt, Germany) or Sigma-Aldrich (St. Louis, MO, USA). 8HD is a gift from Professor T.S. Chang. 8HD was isolated from soy germ koji fermented by A. oryzae BCRC 32288 (Food Industry Research and Development Institute, Hsinchu, Taiwan) in the lab of Professor T.S. Chang. The structure has been identified as 7,8,4′-trihydroxyisoflavone (Figure 1B) [15,16].

### 3.2. Cell Culture

Caco-2 cell line was obtained from the Food Industry Research and Development Institute. Caco-2 cells were cultured in Dulbecco’s modified Eagle’s medium (DMEM, Gibco) supplemented with 10% fetal bovine serum (Hyclone) and 1% penicillin and streptomycin (Hyclone) at 37 °C in a humidified atmosphere of 5% CO_2_ and 95% air.

### 3.3. Cell Growth Inhibition Assay and Isobologram Analysis

Caco-2 cells were seeded in 96-well plates at a density of 3.5 × 10^4^ cells/well, allowed to attach overnight, and then treated with different Epi concentrations with or without 8HD (0, 10, 25, 50, and 100 μM). After 48 h incubation, 100 μL of MTT (0.2 mg/mL) was added to each well, and the cells were reincubated for an additional 4 h. Dimethylsulfoxide (100 μL) was added to each well to dissolve the formazan. Absorbance (OD_545_) was measured at 545 nm using a microplate reader (MRX; Dynatech Laboratories, Chantilly, VA, USA). Cell viability (%) was calculated by dividing the number of cells incubated with Epi and/or 8HD by the number of cells incubated with only DMEM (control). Potency of single administration of Epi, as well as that of combined Epi and 8HD, on cell-growth inhibition is expressed as IC_50_, defined as the drug concentration necessary to inhibit cell growth by 50%. Data are the means ± standard deviation (SD) of three experiments.

In addition, the combination index (CI) in isobologram analysis was used to evaluate the synergistic, antagonistic, or additive effects of the combined treatment of 8HD and Epi. The CI is calculated using the formula: CI = a/A + b/B, in which “a” is the concentration of Epi required to achieve 50% growth inhibition in the combined treatment; “A” is the concentration of Epi that produces an identical effect alone; “b” is the concentration of 8HD which reaches a 50% growth inhibition in the combination; “B” is the concentration of 8HD that causes the same effect alone [46]. CI < 1 represents synergy, CI = 1 stands for additivity, and CI > 1 corresponds to antagonism [47].

### 3.4. Determination of Intracellular Hydrogen Peroxide and Superoxide Oroduction

Cells were seeded in 6-well plates at a density of 2 × 10^5^ cells/well, allowed to attach overnight, and exposed to 0.3 μg/mL of Epi and/or 25 μM of 8HD for 48 h. Cells were then incubated in the dark with DCFH-DA (20 μM) or DHE (5 μM) at 37 °C for 1 h. The cells were then harvested and immediately analyzed using a flow cytometer (Cell Lab Quanta SC MPL (abbreviated as Quanta SC); Beckman Coulter, Fullerton, CA, USA). Data acquisition and analysis were performed using commercial software (Quanta SC) [40].

### 3.5. Real-Time PCR of P-gp, MRP1, MRP2, Bax, Bcl-2, Caspases, and p53

Cells were pretreated with or without the antioxidant NAC (5 mM) for 30 min, and exposed to Epi (0.3 μg/mL) and/or 8HD (25 μM) for 48 h. Total RNA was extracted from cells using the Total RNA Extraction Miniprep System (Viogene, Taipei, Taiwan) according to the manufacturer’s instructions. RNA yield and purity were assessed using *NanoDrop* 2000 (Thermo, Wilmington, DE, USA). cDNA was prepared from total RNA using a High-capacity RNA-to-cDNA kit (Applied Biosystems; Foster City, CA, USA), following the manufacturer’s protocol. Gene-specific primers (Table 1) of MDR1, MRP1, MRP2, Bcl-2, Bax, and p53, as well as caspases-3, -8, and -9, were developed through multiple sequence alignment. GAPDH served as an internal control. Quantitative PCR was performed using the StepOne Real-Time PCR system (Applied Biosystems) and SYBR Green PCR Master Mix (Applied Biosystems). The cycling program was set as follows: denaturation at 95 °C for 10 min, followed by 40 cycles of 95 °C for 15 s and 60 °C for 1 min. Results were normalized to GAPDH level. mRNA expression ratio was calculated as the mRNA expression level compared with the cell control. Each experiment was performed in triplicate.

### 3.6. Measurement of Intracellular Epi Accumulation

Cells were seeded in six-well plates at a density of 2 × 10^5^ cells/well, allowed to attach overnight, pretreated with 25 or 50 μM of 8HD for 1 h. Then, 0.3 μg/mL of Epi was added to the culture medium for 3 h. The cells were collected and suspended in PBS at 37 °C. Flow cytometric analysis was then conducted using a flow cytometer (Quanta SC) equipped with an argon ion laser and operated at 488 nm. At least 10,000 cells were analyzed in each sample. Within each experiment, determinations were performed in triplicate [5].

### 3.7. Detection of Mitochondrial Membrane Potential

Cells (2 × 10^5^ cells/well) were incubated for 48 h with or without 0.3 μg/mL of Epi and 25 μM of 8HD. The cells were then treated with DiOC_6_ (10 μM) for 1 h at 37 °C. Then cells were harvested and immediately analyzed using a flow cytometer (Quanta SC). DiOC_6_ was excited at 488 nm, and fluorescence was analyzed at 525 nm (FL-1) after logarithmic amplification [31].

### 3.8. Determination of Cell Cycle

Cells at a density of 2 × 10^5^ cells/well were incubated for 48 h with or without 0.3 μg/mL of Epi and 25 μM of 8HD. Cells were harvested by centrifugation and fixed gently with 70% of ice-cold ethanol overnight at −20 °C. The cells were collected by centrifugation. Then, the cells were stained with 1 mg/mL propidium iodide (PI), incubated at 25 °C for 30 min in the dark, and analyzed using a flow cytometer (Quanta SC).

### 3.9. Detection of DNA Fragmentation

DNA fragmentation was detected by agarose gel electrophoresis. 2 × 10^5^ cells/well were gently scraped and harvested by centrifugation after Epi and/or 8HD treatments for 48 h. The cells were lysed with lysis buffer (50 mM Tris-HCl, 10 mM EDTA, 0.5% Triton X-100, 0.5 mg/mL Proteinase K) and then incubated at 56 °C for 24 h. DNA was extracted with phenol, chloroform, and isoamyl alcohol (25:24:1), prior to electrophoresis on 2% agarose gel at 50 V. The gel was stained with SYBR^®^ Safe (Invitrogen, Carlsbad, CA, USA) and digitally photographed using a gel documentation system (UVIdoc; UVItec Limited, Cambridge, UK).

### 3.10. Caspases-3, -8, and -9 Activity Assay

The activities of caspases-3, -8, and -9 were detected with Caspase-Glo 3, Caspase-Glo 8, and Caspase-Glo 9 Assay Kits (Promega, Madison, WI, USA), respectively. Cells (2 × 10^5^ cells/well) were harvested after treatment with 0.3 μg/mL of Epi and/or 25 μM of 8HD for 48 h. Fifty microlitres of the cell suspension was then mixed with 50 μL of caspase-3, -8, and -9 reagents containing the corresponding luminogenic substrate of Ac-DEVD-pNA, Ac-LETD-pNA, and Ac-LEHD-pNA, respectively, at room temperature for 30 min. Released aminoluciferin luminescence levels were detected using a luminometer (MiniLumat LB9506; Berthold, Bad Wildbad, Germany) [48].

### 3.11. Statistical Analysis

All data are expressed as means ± SD. Student’s *t*-test was used to analyze differences between two treatment groups. Statistical analysis was also conducted using one-way ANOVA and Dunnett’s multiple comparison tests. Significant difference was set at *p* < 0.05.

## 4. Conclusions

Taken together, the present study pioneers in showing that 8HD and Epi triggers cell death in human colon cancer cells through the ROS-mediated inhibition of P-gp and MRPs, as well as p53 activation of the mitochondrial and death receptor pathways of apoptosis. Hence, simultaneous suppression of pump and non-pump resistance with 8HD and Epi may provide a new strategy to antagonize MDR. 8HD, found in the fermented products of soygerm, intensifies the cytotoxicity of anticancer drugs and thus has the potential to reduce chemotherapy dosage and corresponding side effects, as well as improve the therapy efficacy.

## Figures and Tables

**Table 1:** Real-time PCR primer sequences.

Primer name	Forward primer sequences	Reverse primer sequences
GAPDH	ATGGGGAAGGTGAAGGTCG	GGGGTCATTGATGGCAACAATA
MDR1	GCTCATCGTTTGTCTACAGTTCGT	ACAATGACTCCATCATCGAAACC
MRP1	GGATCATGCTCACTTTCTGG	AAGTGATGTCACGAAACAGGTC
MRP2	AAGATGCAGCCTCCATAACCA	TGGACCTAGAACTGCGGCTAA
p53	GAGAATCTCCGCAAGAAAGG	CTCATTCAGCTCTCGGAACA
Bcl-2	CTTGACAGAGGATCATGCTGTAC	GGATGCTTTATTTCATGAGGC
Bax	GGGCCCACCAGCTCTGA	CCTGCTCGATCCTGGATGA
Caspase-3	CCTGGTTATTATTCTTGGCGAAA	GCACAAAGCGACTGGATGAA
Caspase-8	CAGGCAGGGCTCAAATTTCT	TCTGCTCACTTCTTCTGAAATCTGA
Caspase-9	TGCTGAGCAGCGAGCTGTT	AGCCTGCCCGCTGGAT
